# Supplementary material for: Are carbon emissions trading and green financial instruments synergistic? -Comprehensive quantitative research based on content analysis
Source: PLoS One. 2024 Mar 7;19(3):e0298601. doi: 10.1371/journal.pone.0298601 (PMC10919601; doi:10.1371/journal.pone.0298601)
Supplement: S1 Data — (ZIP) [file pone.0298601.s001.zip › Data/地级市Z_F工作报告.zip!/░▓╗╒/░▓╟∞╩╨/└┤╫╘╣1⁄2╓┌║┼í╛┬φ┐╦╩2╛▌═°í┐_2016.docx]

——2016年1月23日在安庆市第十六届人民代表大会第五次会议上

市长 魏晓明

各位代表：

现在，我代表市人民政府，向大会报告政府工作，请予审议，并请市政协委员和其他列席人员提出意见。

一、“十二五”时期工作回顾

“十二五”特别是党的十八大以来，全市上下在中共安庆市委的坚强领导下，深入贯彻落实党的十八大、十八届三中、四中、五中全会和习近平总书记系列重要讲话精神，积极适应经济发展新常态，坚持稳中求进工作总基调，大力推进“三个强市”建设，“十二五”规划主要任务基本完成，经济社会发展取得令人鼓舞的崭新成就。

——过去五年，经济平稳较快发展。预计2015年地区生产总值超过1600亿元，是“十一五”末的1.6倍。固定资产投资超过1500亿元，社会消费品零售总额超过650亿元，进出口总额超过20亿美元，分别是“十一五”末的1.9倍、1.9倍和2.9倍。财政收入258.8亿元，是“十一五”末的2.1倍；税收收入占比88.1%，比“十一五”末提高2.6个百分点。三次产业结构为13.4∶51.1∶35.5，三产比重比“十一五”末提高3.8个百分点。**工业化进程明显加快。**完成工业投资3350亿元，是“十一五”的2.7倍。石化800万吨炼化一体化、泰发新能源、申洲纺织等一批重大项目建成投产。规模以上工业企业1784家，增加值超过700亿元，是“十一五”末的2倍。战略性新兴产业产值433亿元，是“十一五”末的2倍。新增中国驰名商标26件，总量居全省第二。**服务业不断提升。**新增银行业金融机构8家，贷款余额1432亿元，是“十一五”末的2.6倍。欧尚、家乐福、大润发投入运营，合肥“安庆之窗”重新开张。获批创建“宽带中国”示范城市。入选全国物流园区发展规划布局城市。天柱山成为国家5A景区和世界地质公园，新增国家文化产业示范基地2个、4A景区14个。**农业产业化稳步推进。**“5211”工程付诸实施，优质粮油等5个“百万基地”建设加快，海亮集团、浙商集团现代农业项目建成，农产品加工业产值超过1100亿元，居全省第二。桐城国家现代农业示范区获批，望江经济开发区成为国家农业产业化示范基地。国家级农业产业化龙头企业6家、省级107家，总量全省第一。耕地流转率49.7%，全省领先。农民专业合作社5099家、家庭农场2400家，均居全省前列。去年粮食总产268.3万吨。新增国家地理标志保护产品6个，总量居全省第三。**创新发展能力增强。**大力实施科技创新“一招鲜”工程，与中科院、清华大学等30家名校名所合作，建成实体研究院24家、院士工作站5家、博士后工作站5家，国家石化（安庆）、高分子材料（桐城）质检中心投运。国家农业科技园区获批。中国科协“海智计划”安庆工作基地挂牌。华茂集团获得中国质量奖提名。专利申请量增幅全省领先。高新技术产业增加值超过120亿元，是“十一五”末的3.1倍。

——过去五年，城乡面貌明显改观。城镇建设管理力度加大。新建、改扩建机场大道、中山大道等50条骨干道路，人民路步行街建成开放，城区整治改造背街后巷160条、积水点48处、菜市场31个、临时停车场27个、公厕58座，新建雨水管网335公里、污水管网220公里。市及八县（市）体育场馆、市客运中心站投入运营。积极创建全国国土资源节约集约模范市，6.6万亩批而未供、闲置和低效利用土地处置到位，建成标准化厂房500万平方米。实施市容市貌、违法建设、道路交通等专项整治，蝉联省级文明城市，全面启动全国文明城市创建。加强县城和中心镇建设，夯实产业基础，推进产城一体，增强人口城镇化承载能力。城镇化率43.5%，比“十一五”末提高6.7个百分点。美好乡村建设成效明显。建成省级中心村216个，新建村级公路1400公里，改造农村危房11.2万户，潜山成为全国美好乡村综合标准化示范县，岳西、潜山、桐城评为全省美好乡村建设先进县。完成346个小型病险水库除险加固，解决137.3万农村人口饮水安全问题。建成高标准基本农田177万亩。加强扶贫开发，减少贫困人口78万。综合交通体系不断完善。宁安高铁开通运营，安庆火车站改造完成。岳武高速、望东长江大桥北岸接线建成通车，实现“县县通高速”。改造国省道360公里，完成县乡公路升级1126公里，加固危桥720座。推进城区码头外迁，长风港一期、石门湖航道整治工程完工。天柱山机场民航设施改造完成，年旅客吞吐量首次突破20万人次。

——过去五年，市场活力显著增强。重点领域改革全面深化。完成新一轮政府机构改革任务，市政府部门由39个精简为31个。实施商事制度改革，登记企业超过4.6万家，是“十一五”末的2.2倍。创建全省“审批项目最少、办事效率最高、行政成本最低”城市，权力清单、责任清单、涉企收费清单制度全面实行，市级行政审批项目精简50%，减免行政事业性和经营服务性收费4亿元。深化城区财政体制改革，实行全口径预决算，“营改增”顺利推进。投融资改革力度加大，市级政府平台融资519.9亿元，是“十一五”的5.4倍。农村合作金融机构全部改制为商业银行，村镇银行实现县域全覆盖。6个县（市）整县开展土地承包经营权确权登记颁证试点工作。国家级桐城经济开发区获批。开放型经济加快发展。实现与长江经济带区域通关一体化，开通直达上海集装箱精品航线。组织企业赴“一带一路”沿线拓展合作，国际友好城市发展到8个。建成皖西南公共型保税仓库，获批建设B型保税物流中心。安庆港成为国家进境粮食指定口岸，港口货物吞吐量4002万吨、集装箱5.1万标箱，分别是“十一五”末的1.4倍和3.5倍。招商引资成效明显。加大承接产业转移力度，出台招商引资导则，每年举办北京、上海、广州经贸招商活动，千名干部驻外招商，中船马克柴油机、中建材新能源材料、瑞沃科技产业园等项目建成投产。引进亿元以上项目953个，其中10亿元以上项目116个，利用市外资金是“十一五”的2倍。

——过去五年，人民生活持续改善。民生工程深入实施。民生支出占财政支出比重84.9%，比“十一五”末提高8.4个百分点。城镇、农村常住居民人均可支配收入分别达到24100元、9930元，是“十一五”末的1.8倍和1.9倍。新建保障性安居工程住房11.3万套、基本建成4.7万套。2143户以船为家渔民上岸安居。城区创新实施以“房票”为主的货币化安置政策，真正实现“先安置、后征迁”，棚户区改造工作得到广大群众支持。新增城区至海口镇等15条公交线路，城区乡镇公交基本实现全覆盖。物价保持稳定。社会保障提标扩面。城镇新增就业24.2万人，农村劳动力转移就业32.9万人，城镇登记失业率控制在4.2%以内。城乡居民养老、城镇基本医疗、大病补充保险制度全面实施。城市低保、农村低保和五保供养标准提高，分别是“十一五”末的1.8倍、2.7倍和2.5倍。健全“救急难”机制，在全省率先建立农民工工资专用账户管理机制。生态环保切实加强。成功创建国家森林城市，全国绿化模范城市创建通过验收，全市森林覆盖率37.1%，城区绿化覆盖率43.5%。大力创建国家环境保护模范城市，花亭大沟治理、康熙河水系整治、石化脱硫脱硝改造等重点工程完工。大气污染防治扎实有效，淘汰黄标车和老旧车25743辆，关停超标排放企业38家，空气质量居全省前列。岳西成为国家级生态县，潜山、宜秀成为省级生态县（区）。花亭湖、菜子湖、嬉子湖国家湿地公园获批。节能减排任务完成。社会事业全面进步。出台“1+4”公共服务政策，加大政府购买服务力度，实施70岁以上生活困难老人居家养老服务等项目。国家中小学教育质量综合评价改革实验区建设扎实推进，义务教育“零择校”、教师和校长跨校交流付诸实施，新建、改扩建幼儿园289所，基本完成义务教育学校标准化建设。岳西、大观、迎江、宜秀、桐城成为全国义务教育发展基本均衡县（市、区）。安庆师范学院更名通过评审，黄梅戏艺术职业学院、桐城师范高等专科学校创建成功。国家公共文化服务体系示范区建设深入推进，成功举办第六届、第七届中国（安庆）黄梅戏艺术节，市文化馆、美术馆新馆投入使用，公共图书馆总分馆体系运行，建成社区文化舞台265个、农民文化乐园90个，乡镇综合文化站实现全覆盖。圆满承办省第十三届运动会、第六届残运会和第七届民族运动会，全民健身运动广泛开展。医药卫生体制改革扎实推进。开展全国养老服务业综合改革试点，新建居家养老服务站150个、农村幸福院491个。社会治理不断强化。领导干部开门接访、带案下访、主动约访，信访形势稳定好转。实施“九大安全工程”，安全生产事故起数、死亡人数持续下降。安庆石化油气输送管线迁建工程开工。深化“平安安庆”建设，加强社会治安综合治理，应急管理工作全省先进，群众安全感指数居全省前列。完成“六五”普法任务。国防动员、人民防空工作扎实推进，武警水电、森林部队驻防。森林防火、秸秆禁烧工作取得明显成效，殡葬改革平稳运行。民族宗教、外事侨务、涉台、妇女儿童、老龄、残疾人、红十字等事业进一步加强，气象、地震、防灾减灾等工作取得新进展。

——过去五年，政府自身建设不断加强。践行群众路线，落实八项规定，坚持“三严三实”，坚决反对“四风”。法治政府建设扎实推进，政府法律顾问制度全面实施。自觉接受市人大及其常委会法律监督和市政协民主监督，建立市政府与市人大常委会、市政协联席会议制度，市人大代表议案建议、市政协提案办复率100%。健全重大事项决策机制，土地、规划、国资、投融资等机制逐步完善，招投标和公共资源交易进一步规范，建设管理“六分开”、公益性公用工程“四到位”全面实行。组织群众公议行政处罚案件338件。深化政务公开，强化行政监察、审计监督，加强权力运行监管。加大廉政建设和效能建设力度，政府执行力和公信力进一步提升。

刚刚过去的2015年，面对前所未有的经济下行压力，我们围绕稳增长、调结构、促改革、惠民生、防风险，重点做了五个方面工作：一是扶持实体经济。强化“四大政策”，实施支持中小微企业健康发展20条措施，设立转贷应急资金池。广泛开展“千名干部驻企帮扶”活动。推动困难企业兼并重组，舒美特化纤、通显新材料、稼仙米业等一批企业重获生机。二是发展新兴产业。市高新区成为全省首批战略性新兴产业集聚发展基地。发展新能源汽车产业，江汽轿车项目主体完工，安达尔特种汽车即将下线。提升装备制造产业，富士康自动化设备制造扩能项目投产、智能制造研发量产项目落地。培育电子信息产业，酷米填补全省自主品牌手机空白。发展电子商务产业，智慧产业园成为国家电子商务示范基地，桐城、岳西获批国家电子商务进农村综合示范县，纳入统计的电商企业交易额27.2亿元、增长57.3%。三是加强投融资工作。争取债券资金80.5亿元，获批国家专项建设基金68亿元。市城投公司16亿元企业债券获准发行、40亿元中票成功注册。市经开区“两区共建”项目融资授信100亿元，首期放贷30亿元。新增上市辅导备案企业8家、“新三板”挂牌企业6家。深化政银担合作，国有政策性融资担保机构实现县（市）域全覆盖。与徽商银行合作建立城镇化一号基金，规模达到123.8亿元。全国首例公益类设施PPP项目外环北路开工，城区污水收集处理厂网一体化项目签约，宿松沿江一级公路、怀宁“三馆一中心”等项目开工。四是加快基础设施建设。合安高铁开工。合安高速安庆互通迁建实施。下浒山水库工程开工并实现河道截流。安庆电厂2台百万千瓦超超临界机组并网发电。安庆新机场规划选址完成。市一水厂迁建、大寨沟清淤、潜江路一期等工程顺利完工，外环西路、市博物馆开工。城区至周边县城3条一级公路建设进展顺利。红光、狮子山公交枢纽站动工兴建，首条纯电动公交线33路开通。城区开发园区“五化”建设全面推开。五是开展精准扶贫试点。大别山革命老区振兴规划启动实施，出台“百人供养、千家脱贫、万户增收”扶贫计划，干部驻村帮扶工作得到国务院扶贫办肯定，岳西入选国家首批光伏扶贫试点，完成150个村“整村推进”工程。

各位代表、各位委员、各位同志！五年的成绩来之不易，这是省委、省政府和市委坚强领导的结果，是市人大、市政协和社会各界监督支持的结果，是全市人民共同奋斗的结果。在此，我代表市人民政府，向全市广大工人、农民、知识分子、干部、离退休老同志，向驻宜解放军指战员、武警官兵和政法干警，向各民主党派、各人民团体和各界人士，向关心、支持安庆发展的中央和省驻宜单位、海内外友好人士，向在我市创业的投资者、建设者，表示衷心的感谢！

在肯定成绩的同时，我们清醒地认识到：我市经济在全省总量靠前、人均居后，发展质量、产业层次、创新能力有待进一步提高；基础设施建设仍有不少欠账；县域经济整体实力不强，贫困面大、贫困程度深，脱贫攻坚任务繁重；基本公共服务供给不足，与人民群众期待和愿望还有差距；环境保护、安全生产、征地拆迁等方面还存在一些矛盾和问题；少数干部开拓意识不强，担当负责精神不足，能力水平有待提升。我们将直面问题，逐步解决。

二、“十三五”时期目标任务

未来五年，我市仍然处于工业化、城镇化加速发展阶段，正是大有作为的战略机遇期。我们必须进一步坚定发展信心，强化机遇意识，树立底线思维，主动适应、把握和引领经济发展新常态，奋力把安庆发展推向新的更高水平。

“十三五”发展指导思想是：深入贯彻党的十八大、十八届三中、四中、五中全会和习近平总书记系列重要讲话精神，按照“五位一体”总体布局和“四个全面”战略布局，坚持发展是第一要务，强化创新、协调、绿色、开放、共享五大发展理念，打好产业转型升级、棚户区改造、精准脱贫、重大基础设施建设、增强县域经济实力五大攻坚战，不断提高发展质量和效益，加快建设“四个强市”，确保2020年全面建成小康社会。

发展目标是：

——建设经济强市。经济总量稳居全省前列，生产总值突破2400亿元，财政收入突破400亿元，固定资产投资累计突破1万亿元，常住居民人均可支配收入突破25000元。

——建设创新强市。全面推进体制、科技、管理和文化创新，切实增强企业自主创新能力、科技成果转化能力。高新技术产业增加值占规模以上工业比重力争达到30%，研究与试验发展经费占生产总值比重力争达到2%。

——建设文化强市。争创全国文明城市。全面建成国家公共文化服务体系示范区，大力推进黄梅戏“一唱两走”，提升黄梅戏艺术影响力。文化产业增加值占生产总值比重力争达到5%以上。

——建设生态强市。生态文明体系基本建立，节能减排任务全面完成，大气、水和土壤环境质量总体改善，生态产业发展水平不断提高，力争成功创建国家环境保护模范城市。

主要任务是：

一是打好产业转型升级攻坚战，提升经济发展质量和效益。培育壮大新能源汽车、新材料、智能制造、新一代信息技术等战略性新兴产业，实施“互联网+”行动计划。运用新技术、新模式改造传统产业，形成炼油与化工、纺织服装、食品加工三大千亿产业。大力发展现代物流、金融保险、电子商务等产业，全面提升健康养老、文化旅游等产业，推动房地产业持续健康发展。着眼增强企业核心竞争力，实施工业强基工程，开展质量品牌提升行动，加强科技创新，推进企业上市，培育一批百亿企业。不断加强企业家队伍建设，大力引进创新创业人才团队。

二是打好棚户区改造攻坚战，提高城市规划建设管理水平。坚持统筹规划、连片改造、系统开发，加快棚户区、老旧小区、城中村改造步伐。用五年时间，改造完成城区700万平方米棚户区、255万平方米老旧小区，县（市）改造完成250万平方米棚户区。加强中心城市水、电、路、气、热和地下管廊等公用设施建设，完善商业、教育、文化、体育、公交等公共服务配套，保护历史文化街区，健全污水、垃圾全收集全处理体系，完成河湖连通、水环境治理工程，着力建设宜居、宜业、宜游城市。

三是打好精准脱贫攻坚战，着力保障改善民生。按照“三年脱贫、两年巩固”要求，统筹推进发展生产、易地搬迁、生态补偿、发展教育、社会保障兜底，精准施策、提升实效，确保现行标准下农村贫困人口全部脱贫、贫困村全部出列、贫困县全部摘帽。推进基本公共服务均等化，完善创业扶持政策，促进充分就业。普及十五年基础教育，完善现代职业教育体系，支持高等教育发展。落实全民参保登记计划，加强困难群体基本生活保障。深化医药卫生体制改革，提升群众健康保障水平。落实国家生育政策，促进人口均衡发展。拓宽居民收入增长渠道，确保居民收入增幅不低于生产总值增幅。

四是打好重大基础设施建设攻坚战，增强区域发展支撑力。建成“十字型”高铁骨架，推进“米字型”铁路网络建设，打造成为全省重要的铁路交通枢纽。提升长江和内河航道等级，推进安庆新港规划建设，把安庆港打造成江海联运枢纽、上海国际航运中心重要喂给港。加密高速公路网络，改造提升国省道，畅通农村道路，打造承东启西、连南接北的交通节点城市。力争建成安庆新机场。着眼基本解决洪涝灾害问题，建成下浒山水库，推进华阳河蓄滞洪区安全建设等重大水利工程。

五是打好增强县域经济实力攻坚战，形成加快发展新格局。坚持彰显特色，提升质量效益，奋力在全省县域发展中争先进位。做大做强支柱产业，培育特色优势，提高产业层次和水平。加强县城和中心镇规划，完善公共设施，健全公共服务体系，促进产城一体。推进一二三产融合发展，建成5个“百万基地”，打造种养加销全产业链，提升农业现代化水平。全面推进美丽乡村建设，优化农村人居环境。

三、2016年重点工作

今年是全面建成小康社会决胜阶段的开局之年，也是推进结构性改革的攻坚之年。我们要坚持改革开放，坚持稳中求进工作总基调，坚持稳增长、调结构、惠民生、防风险，在适度扩大总需求的同时，着力加强供给侧结构性改革，统筹抓好去产能、去库存、去杠杆、降成本、补短板，增强持续增长动力，保持经济社会平稳较快发展，努力实现“十三五”发展良好开局。

基于发展动力正在转换，面对经济下行压力，今年经济社会发展的主要预期目标是：生产总值增长8%，财政收入同步增长，固定资产投资增长12%，社会消费品零售总额增长11%，进出口总额增长不低于全省平均水平，城镇、农村常住居民人均可支配收入分别增长8%和8.5%，居民消费价格涨幅3%左右，城镇登记失业率控制在4.2%以内，确保完成节能减排任务。

重点做好九个方面工作：

（一）实施“调转促”行动

加快工业转型升级。集中力量推动新能源汽车、新材料、智能制造、新一代信息技术等产业发展。完成投资800亿元以上，新增规模以上工业企业150家以上。加强化工新材料产业集聚发展基地建设。加快新能源汽车、赛富环新汽车零部件产业园建设，形成年产5万辆轿车、2万辆特种用车整车生产能力。建成富士康安庆研发中心，加快智能制造基地、中船海工装备项目建设，建成晨兴机器人项目。加快南方、宝捷讯、卡为智能电子科技产业园建设，促进电子信息产业集群发展。推进桐城、岳西抽水蓄能电站项目前期工作，开工建设潜山500千伏变电站。实施100项亿元以上重点技术改造项目，增强企业核心竞争力。积极引进战略投资者，推动企业兼并重组，培育一批骨干企业。支持民营经济发展，促进“专精特新”中小微企业成长。开展降低企业成本专项行动，简政放权，减税降费，更好服务市场主体。

提升现代服务业。发展研发设计、科技服务、人力资源服务等产业。吸引股份制银行来宜设立分支机构，规范发展担保、小贷、典当等类金融机构，严厉打击非法集资和恶意逃废债行为。推动光彩大市场升级改造，建成新城吾悦、八佰伴、百联等城市综合体，推动林安物流等项目落地。开工建设市会展中心。城区体育场馆实行市场化运营。争创国家电子商务试点城市，推动电商进农村、进企业、进社区，培育年销售收入超千万元电商企业30家以上。支持房地产业健康发展，优化投资和供给结构，深化与养老、物流、文化、旅游等产业融合发展，培育房地产租赁市场。推进新市民公积金归集扩面，提升住房消费能力。

推进农业产业化。稳定粮食生产。深入实施“5211”工程，新增绿色有机农产品基地10万亩。培育新型市场主体，新增农民专业合作社200家以上、省级农业产业化联合体10家以上、省级示范家庭农场30家以上。办好上海、合肥农展，启动市农产品综合批发大市场建设。争创怀宁国家现代农业示范区，加快安庆海峡两岸农业合作试验区创建。

加强科技创新。建设产业共性技术研发平台和实体研发机构10家，实现众创空间等新型孵化器县域全覆盖，促进大众创业、万众创新。加强知识产权保护，支持企业自主创新，确保专利申请、专利授权总量全省前列。发展科技金融，吸引天使、风投、创投基金入驻。深入实施“人才强市”战略，大力引进创新创业人才团队，新认定国家高新技术企业50家。加强“质量安庆”建设，争创中国驰名商标2件、安徽著名商标40件、安徽名牌10个。

（二）加强中心城市规划建设管理

推进老城改造、新城配套。建成外环北路、潜江路，改造湖心中路、迎宾西路。推广“房票”安置办法，改造棚户区150万平方米以上，整治老旧小区8个，基本完成背街后巷改造。实施老城区“五化”工程，建设一批游园、绿地，提升市民生活舒适度。建成合安高速安庆互通迁建工程，开工建设城区污水收集处理厂网一体化、静脉产业园项目，推动河湖连通、水环境治理PPP项目签约。新建停车场10个、充电桩1000根，新建、改建一批公厕。巩固全国国土资源节约集约模范市创建成果。启动城市轨道交通建设规划编制。加强历史文化街区保护。

推动产城一体。加快市经开区“两区共建”项目建设，完成8.4平方公里征地拆迁工作，基本形成路网框架。建成市高新区凤凰片公共管廊、110千伏供电线路，改造城西污水处理厂，启动山口片基础设施建设，创建国家循环经济工业园。继续推进城区老工业区搬迁改造。

深化全国文明城市创建。推进“六城同创”。全面整治占道经营、夜市烧烤点、流动摊群点。规范交通秩序，严查交通违法行为，整治车辆乱停乱放。强化广告设置管理。加强社会主义核心价值观宣传教育，深化窗口行业文明创建，提升市民素质，广泛开展群众性创建活动。

（三）推动县域加快发展

加强县城、中心镇和开发园区建设。按照城市标准规划、建设、管理县城，统筹各类空间规划，推行“多规合一”。做优做特城镇产业，打造一批工业重镇、农业强镇、商贸大镇和旅游名镇。全面加强开发园区承载能力和主导产业建设，不断加快工业化、城镇化步伐。

强化交通、水利等基础设施建设。提升县县连通公路等级，实施农村道路畅通工程，完成3000公里改造提升任务。加快下浒山水库建设步伐。全面推进“5588”小型水利工程建设，除险加固小水库47座，建成高标准基本农田16万亩，巩固提升农村饮水安全工程成果。

推进美丽乡村建设。全面启动美丽乡镇建设，大力治脏、治乱，加强基础设施建设和公共服务配套，完成64个乡镇政府驻地建成区环境整治。实施86个省级中心村、一批市县级中心村建设，开展自然村环境整治，实现由点到面、全面推开。支持潜山整县推进美丽乡村建设。

开展脱贫攻坚。积极争取国家和省扶贫开发政策支持，实现375个贫困村“整村推进”工程全覆盖。每个贫困村重点发展1个特色产业，有生产能力的贫困户落实脱贫项目。实施96个贫困村、1万户贫困户光伏电站建设。推进易地扶贫搬迁工程，覆盖7800户、2.4万人。支持岳西在全省率先脱贫。力争127个贫困村脱贫出列，减少贫困人口15万以上。

（四）加快骨干交通工程建设

开工安九高铁项目，推进六安景、随麻安、武安杭等铁路项目前期工作。深化与中远集团合作，改造提升五里庙集装箱码头，启动长风港二期建设，建成石门湖工业品码头，加快安庆新港规划建设。推动长江深水航道向上延伸。建成望东长江大桥，推动岳武高速东延段等项目，继续推进城区至桐城、望江等一级公路建设。加快安庆新机场、天然气绕城环线等项目前期工作。

（五）促进文化繁荣发展

完善公共文化服务。建成市博物馆，推进基层综合文化服务中心全覆盖。加强黄梅戏等文化精品生产，办好“黄金周·黄梅戏”展演活动，推进文化下基层，实现文化惠民乐民。确保国家公共文化服务体系示范区创建通过验收。

不断壮大文化旅游产业。挖掘传统文化底蕴，嫁接现代科技元素，大力发展新业态、新模式，建设市文化创意产业孵化基地。开展省级健康旅游基地创建工作，加强休闲度假、自驾游营地等基础设施建设，推动天柱山旅游公司战略重组，支持五千年文博园创建5A景区，深化“宜游宜秀”建设。推进大别山（安庆）国家旅游应急救援中心规划建设。

（六）强化生态环保建设

深入实施环保创模工程。深化大气污染防治专项行动，做好淘汰黄标车、秸秆禁烧等工作，确保空气质量全省前列。加强水污染防治，建成城东污水处理厂二期，完成谷桥大沟治理，加快建设桐城、太湖、岳西等污水处理厂。基本完成石塘湖备用水源建设。支持潜山、太湖、宜秀创建国家级生态县（区）。完成森林增长提质工程任务，探索划定区域生态红线。

完成节能减排任务。加强对涉重、医药、造纸、化工等企业监督管理，严惩环境违法行为。完成安庆电厂减排改造。发展风能、太阳能、生物质能等新能源和可再生能源，推进建筑、交通运输、商业和公共机构等领域节能工作。

（七）全面扩大开放

拓展开放型经济。启动实施“2115”工程，重点培育20家加工贸易、商贸服务龙头企业，借鉴台湾等地经验发展养老、文化、旅游等产业，运用上海等地模式提升城市商业业态，加快建设加工贸易基地、港口物流基地、绿色有机农产品集散地、中部小商品集散地和出口商品重要输出地。建成B型保税物流园区，申报建设进境肉类指定口岸，推动进口商品直销店落地。鼓励环新、华茂等企业跨国并购、产能合作，继续支持中小企业拓展国际市场。

狠抓招商引资。积极参与国际徽商大会，办好北京、上海、广州经贸招商活动，深化“千名干部驻外招商”行动。加强与台湾电电公会等交流合作，举办“台北-安庆”经贸活动。加大对德、日、美等发达国家招商力度，探索建立境外招商平台。发挥市经开区、高新区、桐城开发区主平台作用，在新能源汽车、化工新材料、机器人产业等方面，着力引进一批10亿元以上重大项目。全市引进亿元以上项目200个以上。

（八）不断深化改革

深化国资国企改革。健全国资监管制度体系，完成市级政府平台公司市场化改造。推动华茂集团整体上市。完善国有集体企业改革改制政策。深化财税体制改革。科学界定市、县（市、区）事权和支出责任。完善政府性债务管理制度。全面完成“营改增”，落实税制改革和税收征管体制改革任务。优化财政支出结构，强化经济促进政策，进一步加大对实体经济支持力度。深化投融资机制改革。创新“4321”政银担合作、“税融通”落实机制，市级政府性融资担保公司注资到10亿元，县、区级分别不低于2亿元和1.5亿元，支持民营担保机构规范发展。推广运用PPP模式，吸引社会资本参与公共服务和基础设施建设。加快企业上市步伐，力争1家企业成功上市，新增上市辅导备案企业6家、“新三板”挂牌企业10家。加快国家专项基金项目建设，积极谋划争取后续项目。建立产业发展基金和股权投资基金。深化农村综合改革。完成农村土地承包经营权确权登记颁证任务，推动农村产权交易市场建设，稳妥推进土地承包经营权、农民住房财产权抵押贷款试点。做好第三次全国农业普查工作。深化“放管服”改革。推广“互联网+政务服务”，推行网上行政审批。全面落实“三证合一、一照一码”登记制度。深化户籍制度改革。全面实施居住证制度，推动进城落户农民“三权落实、五有并轨”，加快农民工市民化进程。支持配合司法体制改革。

（九）着力加强社会建设

切实做好民生工作。实施33项民生工程。新增城镇就业4万人以上。全面开展“工学一体”试点，推进产教结合，加强职业技能培训。积极争创国家“公交都市”试点，加强新能源公交线路建设。推进城乡居民基本医疗保险制度整合，开展重特大疾病医疗救助工作。提高城乡低保标准，加强“救急难”工作。建立贫困精神残疾人救治救助专项工作机制，努力做到相对集中收治，尽力给予生活保障支持。加大政府购买服务力度，支持社会组织、市场主体参与公共服务。

加快发展社会事业。完成国家中小学教育质量综合评价改革实验区建设任务。推进城区教育管理体制改革，充分发挥安庆一中、二中、四中、高琦小学、人民路小学、墨子巷幼儿园等示范带动作用，推动名校（园）集团化办学，扩大优质教育资源。全面推行教师、校长交流轮岗。完成第二期学前教育三年行动计划，推进农村教学点在线课堂全覆盖，确保怀宁、潜山、宿松、望江通过全国义务教育发展基本均衡县验收。提升高中教育质量，推进面向市域自主招生，开工建设安庆一中龙山校区。构建现代职业教育体系，以安庆职业技术学院为龙头，整合职教资源，实行联合办学。提升高等教育发展水平，支持安庆师范学院创建高水平应用型大学，推动安庆医专与安徽医科大学合作办学，建设桐城师专新校区。促进民办教育规范健康发展。举办市第十三届运动会和第二届市民运动会。深化医药卫生体制改革，发展医疗服务联合体，推行分级诊疗，鼓励社会办医。建成市立医院新院区，建设市一院龙山院区。落实全面二孩政策，加强优生优育服务。深化养老服务业综合改革，发展养老产业，推进医养结合，新建、改扩建居家养老服务站170个。

不断创新社会治理。开展安全生产执法能力建设年活动，强化队伍建设，充实专业力量，夯实基层基础。推进安庆石化油气输送管线迁建，全面完成铁路道口“平改立”，加强交通、消防、危爆物品、公共卫生、建筑施工等安全管理。积极创建省级食品药品安全城市。做好森林防火、防汛减灾工作。完善社会治安防控体系，健全矛盾纠纷多元化解机制，切实提升应急处置能力。加快社会服务管理信息化建设，完善社区网格化管理。实行“阳光信访”，及时就地依法解决信访问题。深入推进“守护平安”系列行动，全面实施“天网工程”。做好法律援助、人民调解和社区矫正工作。充分发挥工会、共青团、妇联等人民团体作用。做好科普、统计、地震、气象、档案、地方志、外事、侨务、民族、宗教、涉台、老龄、残疾人、红十字、关心下一代、无线电管理等工作。推进军民融合深度发展，支持驻宜部队建设，加强国防教育、国防动员、人民防空和民兵预备役工作，深入开展“双拥”共建活动。

四、加强政府自身建设

决战“十三五”，全面建成小康社会，需要全市上下共同努力，更需要政府自身积极作为。我们将严格遵守政治纪律和政治规矩，切实增强“看齐”意识，坚持做到“四个自觉”，努力建设人民满意的政府。

一要坚持依法行政。自觉接受市人大、市政协监督，广泛听取民主党派、工商联、无党派人士意见，主动接受社会舆论监督。加强政府立法工作，依法合规决策施政，强化综合执法改革，完善行政执法群众公议制度，做好行政复议、应诉工作。深化政务公开，完善权力清单、责任清单、涉企收费清单，探索实施负面清单，努力做到阳光透明。

二要大力改革创新。坚持问题导向，把握结构性改革机遇，积极变中求进，努力弯道超越。全面扩大开放，运用竞争机制，统筹社会资源，实现公共利益最大化。创新工作机制，突破现行体制制约，打破条块分割惯性，推行“专班突击、集中攻坚”等有效模式，整合力量解难题、办大事。鼓励县（市）区、部门互相借鉴、取长补短，推动形成比学赶超、争先进位的工作格局。加强对基层工作指导，支持一线创新创造，为埋头苦干者助力，为担当负责者解难，为改革创新者鼓劲，不断激发干事创业的热情和活力。

三要提高行政效能。开展行政审批改革“回头看”，进一步简化流程，减少收费，提高效率，不断巩固“三最”城市建设成果。倡导专业的人干专业的事，加强干部实践历练和实战锤炼，鼓励急用先学、干中求知，增强推动中高端发展、大规模建设、创新投融资等方面能力。实行闭环管理，加强督查督办，完善绩效考核，严格兑现奖惩。办好“1584”政风行风热线，加强政民互动交流，不回避问题，不掩饰矛盾，做到民有所愿、施政所向。注重工作协调沟通，加强宣传动员，广泛凝聚力量，营造众人拾柴、团结成事的良好氛围。

四要保持清正廉洁。严守八项规定，践行“三严三实”，持之以恒纠正“四风”。严格履行“一岗双责”，立“明规矩”、破“潜规则”，不断压缩腐败现象生存空间和滋生土壤。加强行政监察、财政监督和审计结果运用，坚决查办重点领域、关键环节的腐败案件，认真纠正和严肃查处基层贪腐以及执法不公等问题，推动党风廉政建设落实到基层，维护群众切身利益。

各位代表、各位委员、各位同志，蓝图已经绘就，任务非常明确，实干才有实效，善做更要善成。让我们紧密团结在以习近平同志为总书记的党中央周围，在中共安庆市委的坚强领导下，与全市人民一道，锐意改革，开拓创新，为打造“四个强市”、全面建成小康社会而努力奋斗！
